# Prevalence of arterial stiffness in North China, and associations with risk factors of cardiovascular disease: a community-based study

**DOI:** 10.1186/1471-2261-12-119

**Published:** 2012-12-07

**Authors:** Jin-Wen Wang, Zi-Qiang Zhou, Da-Yi Hu

**Affiliations:** 1 Beijing Anzhen Hospital, Capital Medical University, Beijing Institute of Heart Lung and Blood Vessel Disease, Beijing, 100029, People’s Republic of China; 2 Cardiovascular Center, Beijing Tongren Hospital, Capital Medical University, Beijing, 100730, People’s Republic of China; 3 Heart Center, People’s Hospital of Peking University, Beijing, 100035, People’s Republic of China

**Keywords:** Brachial-ankle pulse wave velocity, Arterial stiffness, Risk factor, Cardiovascular disease

## Abstract

**Background:**

Brachial-ankle pulse wave velocity (baPWV), which reflects the stiffness of both central and peripheral muscular arteries, has been frequently used as a simple index for assessing arterial stiffness. The aim of the present study was to investigate the prevalence of arterial stiffness in North China based on baPWV measurements, and explore the associations between increased arterial stiffness and risk factors of cardiovascular diseases (CVD).

**Methods:**

Twenty-three community populations were established in North China. For each participant, parameters for calculating baPWV, including blood pressures and pressure waveforms, were measured using a non-invasive automatic device. All participants were required to respond to an interviewer-led questionnaire including medical histories and demographic data, and to receive blood tests on biochemical indictors.

**Results:**

A total of 2,852 participants were finally investigated. Among them, 1,201 people with low burden of CVD risk factors were chosen to be the healthy reference sample. The cut-off point of high baPWV was defined as age-specific 90^th^ percentile of the reference sample. Thus, the prevalence of high baPWV was found to be 22.3% and 26.4% in men and women respectively. After adjusted for age, heart rate (HR), systolic blood pressure (SBP), fasting glucose level, and smoking were significantly associated with high baPWV in men; while level of serum total cholesterol (TC), HR, SBP, and diabetes were significantly associated with high baPWV in women.

**Conclusions:**

Based on the age-specific cut-off points, the middle-aged population has a higher prevalence of high baPWV in North China. There exists a difference between men and women in terms of the potential risk factors associated with arterial stiffness.

## Background

Increased arterial stiffness and atherosclerosis are associated with several risk factors of cardiovascular diseases (CVD), such as elevated level of blood glucose, hypertension, obesity, and smoking [1-3]. Pulse wave velocity (PWV), which reflects the stiffness of both central and peripheral muscular arteries, can be calculated via non-invasive measurements. In two previous studies, PWV was used as a simple index for assessing arterial stiffness and atherosclerosis [4,5]. And researches [6,7] have demonstrated good validity and reproducibility of brachial-ankle PWV (baPWV), as well as its strong association with central PWV. During the recent years, baPWV measurements have been carried out in several university hospitals in China due to its convenience and importance. Up to now, however, no epidemiological data on the prevalence of increased arterial stiffness defined by high baPWV in Chinese population have been published. Therefore we designed the present study in order to investigate the prevalence of increased arterial stiffness in North China based on baPWV measurements.

In addition, preliminary studies based on white cohorts have shown that PWV is significantly associated with conventional CVD risk factors, including age, hypertension, hyperglycemia, and hypercholesterolemia [8]. However, association between unconventional risk factors like heart rate (HR) and higher arterial stiffness remains unclear. Furthermore, no study on the associations between arterial stiffness and CVD risk factors based on China’s population has been reported. Consequently, the second aim of this study is to explore the potential CVD factors associated with arterial stiffness defined by high baPWV in Chinese community-based populations.

## Methods

### Participants

The cross-sectional survey was conducted between January and August 2008. Twenty-three community-based populations from Beijing and Hebei Province were chosen by simple random sampling, such that these populations represented the average standards of living, education, and health care in North China. Those (n = 5,770) who were community-dwelling residents aged 18 years or older in these 23 areas, were located on the basis of the local resident registry of each community. Being clearly informed of the study protocol, 3,093/5,770 (53.6%) agreed to participate in this survey. The most common reason for non-participation (46.7%) was due to refusal of blood test or examination. Written informed consent was obtained for each participant, who was required to correspond to an interviewer-led questionnaire with questions on smoking habits, occupation, reproductive history, and medical histories. Trained physicians performed as interviewers. Except for oral reports, detailed information regarding medical histories of all participants was provided by community health service of each community. Approval of this study was granted by the Institutional Review Board, Capital Medical University.

Those (241 of 3,093) who had one or more of the following factors were excluded from this study: a) aortic valve diseases and/or aortic aneurysm, for they are frequently associated with abnormalities of the systemic arterial system, and, in particular, with reduced arterial compliance, which may have important influence on PWV; b) history of percutaneous coronary intervention and/or coronary artery bypass grafting, for these patients may suffer from potential injuries of arteries due to puncture or artery harvesting, which might affect the test result of the automatic device used in the present study; c) ABI of 0.9 or less, for this indicates the probability of obstructive arteriosclerosis, which could lead to a decreased PWV value; and d) ejection fraction < 30%, for patients with severe heart failure tend to have an increased PWV.

Finally 2,852 eligible participants were involved. From these people, a healthy reference sample (n =1,201) was established, for participants of this sample had none of following issues:

1) hypertension (defined as systolic blood pressure [SBP] ≥ 140 mmHg and/or diastolic blood pressure [DBP] ≥ 90 mmHg, and/or antihypertensive drug(s) usage);

2) diabetes (defined as level of fasting blood glucose ≥ 126 mg/dL or receiving insulin therapy and/or taking anti-diabetic medications);

3) dyslipidemia (defined as serum total cholesterol [TC] level > 6.2 mmol/L and/or triglycerides [TG] > 2.3 mmol/L or taking lipid-lowering medicines);

4) CVDs (including coronary heart disease, heart failure, stroke, transient ischemic attack, and/or intermittent claudication);

5) current smoking (defined as smoking within the past twelve months);

6) obesity (defined as body mass index [BMI] ≥  30 kg/m^2^).

### Measurement of baPWV

An automatic device (ModelBP-203PRE, Colin Inc., Komaki, Japan) was used to perform baPWV measurements. Each participant was asked to take five minutes of rest and then to take examinations in the supine position with cuffs wrapped both on brachia and on ankles, electrocardiogram electrodes placed on both wrists, and a microphone for detecting heart sounds placed on the left edge of the sternum. The pulse volume waveforms were recorded via a plethysmographic sensor connected to the cuffs. Volume waveforms for the brachium and ankle were stored, then an automatic gain analysis with quality adjustment would be done after ten seconds’ sampling. Time interval between the brachial and ankle (Tba) was defined as time between the initial rise in the brachial and tibial pressure waveforms. The distance between two sampling points of baPWV could be calculated automatically based on the subject’s body stature. Both length and height were measured to the nearest of 1 centimeter (cm).The length from aortic valve to ankle (La) was calculated via the following equation: La (cm) = 0.8129 × height (cm) + 12.328; while that from the aortic valve to the brachia (Lb) was computed using another equation: Lb (cm) = 0.2195 × height (cm) – 2.074. Finally, based on the equation: baPWV = (La–Lb) / Tba, value of baPWV was obtained. In the meantime, bilateral baPWV, brachial and ankle blood pressures, electrocardiogram, and heart sounds were also obtained via automatically computation by this device. In this study, the mean value of the bilateral baPWV, and left brachial blood pressure were used.

### Laboratory measurements

After 12–15 hours of fasting, a venous blood sample of 10 ml was taken from each subject. The blood samples were preserved in tubes containing 0.1% EDTA. Plasma was isolated by centrifugation at 2500 rpm, 4°C, for 20 minutes and then shipped in the frozen state using dry ice to the central laboratory. Specimens were all stored at −70°C before laboratory assays was carried out. Measurements of high density lipoprotein cholesterol (HDL-C) level complied with that described in Manual of Laboratory Operations of the Lipid Research Clinics Program [9], where the intra-assay coefficient of variation (CV) and inter-assay CV are required to be 1.4% and 2.0% respectively. Levels of TC and TG were measured using standard enzymatic methods [10], where intra-assay CV and inter--assay CV are 0.9% and 1.8% respectively for TC, 1.5% and 1.9% respectively for TG. Levels of fasting glucose were measured using the hexokinase method, where intra-assay and inter-assay CV are required to be 1.9% and 2.6% respectively. For those with TG < 400 mg/dl, low-density lipoprotein cholesterol (LDL-C) levels were calculated using the Friedewald equation [11]: $\mathrm{LDL}-C\frac{\mathrm{TC}-\mathrm{HDL}-C-\mathrm{TC}}{S}$. No participant with TG ≥ 400 mg/dl was found in this investigation. The central laboratory of The Military General Hospital of Beijing PLA was responsible for all the blood tests in this study. The internal and external quality controls procedures were performed in accordance with regulations of Chinese Laboratory Quality Control.

### Statistical analysis

Statistical analyses were performed using the SPSS for Windows statistical software package version 15.0 (SPSS Inc., Chicago, IL, USA). Continuous variables were expressed as mean ± standard deviation (SD) unless otherwise indicated. Differences in continuous variables between the subjects with and without high baPWV were tested with Student’s t test, including age, BMI, SBP, DBP, HR, and levels of TC, HDL-C, LDL-C, TG and fasting glucose. Differences in binomial categorized variables between the two groups were analyzed with Person’s Chi-square test, including hypertension (yes/no), diabetes (yes/no), smoking (yes/no, defined as smoking regularly over the prior 12 months), ischemic heart disease (yes/no), stroke (yes/no), angiotensin converting enzyme inhibitors (ACEIs) and/or angiotensin receptor blockers (ARBs) usage (yes/no), statins usage (yes/no), calcium-channel blockers (CCBs) usage (yes/no). Considering the difference between men and women, we performed multiple analyses in two genders separately. Age-specific cut-off points for high baPWV were defined as the 90th percentiles in each age group of the healthy reference sample respectively.

Multivariate analyses based on multiple backward stepwise logistic regression were also performed to test the associations between potential risk factors and increased arterial stiffness defined as high baPWV, in men and women separately. Variables were considered for entry into the multiple logistic models were age, BMI, HR, SBP, DBP, HR, TC, HDL-C, TG, fasting glucose, diabetes, ischemic heart disease, hypertension, stroke, smoking, statin usage, ACEIs and/or ARBs usage, and CCBs usage. The standard used for a variable entering or not entering the multivariate analysis was based on p < 0.10 yielded by univariate analysis, and/or its potential clinical significance on the basis of previous studies and clinical practice. The criterion for inclusion of variables was p < 0.05, and that for exclusion was p > 0.10. All p values reported are two-tailed and p < 0.05 was considered to be statistically significant.

## Results

A total of 2,852 subjects were finally investigated. The demographic data, disease characteristics, and mean values of biochemical indictors of the study population are shown in Table 1.

**Table 1:** Characteristics of study population

	**Healthy sample**		**Overall sample**	
	**male**	**female**	**male**	**female**
**N**	342	859	1275	1577
**Age , years**	46.2 ± 15.4	46.7 ± 13.8	52.5 ± 12.7	54.0 ± 14.9
**BaPWV, cm/s**	1339.3 ± 224.8	1294.8 ± 241.9	1524.8 ± 355.7	1578.6 ± 463.6
**BMI, kg/m**^**2**^	23.8 ± 2.9	22.8 ± 2.9	24.7 ± 3.2	23.9 ± 3.4
**MAP, mmHg**	89.8 ± 7.3	85.3 ± 8.8	97.9 ± 18.8	94.7 ± 13.3
**SBP, mmHg**	123.9 ± 13.2	120.6 ± 12.1	131.8 ± 17.9	130.9 ± 20.9
**DBP, mmHg**	74.4 ± 7.5	70.2 ± 8.4	80.9 ± 24.1	76.6 ± 10.9
**Pulse pressure, mmHg**	46.0 ± 7.9	45.3 ± 9.1	50.9 ± 24.9	54.5 ± 14.9
**HR, beats/min**	70.8 ± 9.4	71.4 ± 9.4	70.9 ± 10.2	71.6 ± 10.1
**TC, mmol/L**	4.57 ± 0.81	4.75 ± 0.94	4.73 ± 0.94	5.09 ± 1.06
**HDL-C, mmol/L**	1.37 ± 0.29	1.44 ± 0.33	1.29 ± 0.29	1.39 ± 0.33
**LDL-C, mmol/L**	2.67 ± 0.62	2.75 ± 0.83	2.81 ± 0.76	3.02 ± 0.91
**TG, mmol/L**	1.53 ± 1.04	1.31 ± 0.87	1.80 ± 1.27	1.59 ± 0.99
**Fasting glucose, mmol/L**	4.9 ± 0.6	4.9 ± 0.5	5.4 ± 1.4	5.4 ± 1.6
**Hypertension, %**	0	0	50.3	47.8
**Diabetes, %**	0	0	23.7	21.1
**Smoking, %**	0	0	50.2	3.7
**Stroke, %**	0	0	7.2%	6.5%
**Ischemic heart disease, %**	0	0	10.9	7.1
**Medication**				
**ACEIs/ARBs, %**	0	0	9.9	8.9
**Statins, %**	0	0	6.6	4.9
**CCBs, %**	0	0	15.3	14.5

***BMI*** body mass index, ***SBP*** systolic blood pressure, ***DBP*** diastolic blood pressure, ***MAP*** Mean arterial pressure; ***BaPWV*** Brachial-ankle pulse wave velocity, ***CCBs*** Calcium channel blockers, ***ACEIs*** angiotensin converting enzyme inhibitors, ***ARBs*** angiotensin receptor blockers, ***HR*** heart rate, ***TC*** total cholesterol, ***LDL-C*** low density lipoprotein cholesterol, ***HDL-C*** high density lipoprotein cholesterol, ***TG*** triglyceride.

Table 2 shows the age-specific mean values and 90^th^ percentile upper limits for baPWV, defined as cut-off points, in both genders of the healthy reference sample. The cut-off points of baPWV increased substantially with the advancing age in the healthy sample.

**Table 2:** Age-specific cut-off points of BaPWV* in male and female in the healthy reference sample

**Age(n)**	**Mean**		**90**^**th**^**percentile (cut-off)**	
	**male**	**female**	**male**	**female**
**<29 (229)**	1200	1125	1340	1285
**30-39 (375)**	1253	1184	1454	1354
**40-49 (475)**	1309	1241	1520	1412
**50-59 (733)**	1330	1353	1503	1603
**60-69 (556)**	1571	1572	1914	1858
**>70 (484)**	1759	1805	2441	2368

* ***BaPWV*** Brachial-ankle pulse wave velocity.

The sex-specific prevalence of high baPWV was calculated based on cut-off points listed in table 2, and is shown in Table 3. In the study population as a whole, the prevalence of high baPWV was 24.5% (22.30% in men and 26.40% in women). In men, the 50–59 years age group had the highest prevalence of high baPWV (39.6%), while the 60–69 years age group had the highest prevalence of high baPWV (38.8%) in women.

**Table 3:** Prevalence of high baPWV* by age group

**Age (n)**	**Male**	**Female**
**<29 (229)**	23.6%	10.6%
**30-39 (375)**	16.1%	14.2%
**40-49 (475)**	18.8%	25.9%
**50-59 (733)**	39.6%	26.6%
**60-69 (556)**	17.6%	38.8%
**>70 (484)**	11.2%	26.1%
**Overall**	22.3%	26.4%

* ***BaPWV*** Brachial-ankle pulse wave velocity.

The study population was divided into two groups based on the cut-off points of each age group, in men and in women respectively. As shown in Tables 4 and 5, compared with participants with low baPWV, those with high baPWV were significantly older (in men: 52.3 ± 16.2 vs. 53.2 ± 13.6, p < 0.001; in women: 52.8 ± 15.2 vs. 58.1 ± 13.2, p < 0.001), and had a higher HR (70 ± 10 vs. 74 ±11, p < 0.001 in men; and 71 ± 9 vs. 75 ± 11, p < 0.001 in women) and level of fasting glucose (5.3 ± 1.2 vs. 5.9 ± 1.9, p < 0.001 in men; and 5.31 ± 1.5 vs. 5.89 ± 1.9, p < 0.001 in women) than those with low baPWV. Though women with high baPWV had a significantly greater BMI (23.9 ± 3.3 vs. 24.4 ± 3.7, p = 0.026), it was not the case for men (24.5 ± 3.3 vs. 25.5 ± 2.9, p = 0.069). Also, ACEIs/ARBs and CCB usage were both more prevalent in high baPWV group than in low baPWV group, irrespective of sex group. Prevalence of diabetes and hypertension were both higher in those with high baPWV compared with the study population with low baPWV. As for levels of blood lipid indices (TC, TG, HDL-C, LDL-C) tested, no significant difference was found between the two groups in men, while they were higher in high baPWV group in women. Smoking was significantly more prevalent in the high baPWV group than the other in men rather than in women.

**Table 4:** Characteristics of male participants with vs. without high baPWV in the overall sample

**Variables**	Low baPWV (baPWV < cut-off)*	High baPWV (baPWV > cut-off)*	**p value**
**n**	995	281	
**Age , years**	52.3 ± 16.2	53.2 ± 13.6	<0.001
**baPWV, cm/s**	1432 ± 269	1854 ± 424	<0.001
**BMI, kg/m**^**2**^	24.5 ± 3.3	25.5 ± 2.9	0.069
**SBP, mmHg**	128.2 ± 15.7	144.4 ± 19.4	<0.001
**DBP, mmHg**	79.0 ± 26.3	87.6 ± 11.5	<0.001
**HR, beats/min**	70 ± 10	74 ±11	<0.001
**TC, mmol/L**	4.7 ± 0.9	4.8 ± 0.9	0.995
**HDL-C, mmol/L**	1.3 ± 0.3	1.3 ± 0.3	0.870
**LDL-C, mmol/L**	2.8 ± 0.8	2.9 ± 0.7	0.407
**TG, mmol/L**	1.8 ± 1.3	1.9 ± 1.3	0.751
**Fasting glucose, mmol/L**	5.3 ± 1.2	5.9 ± 1.9	<0.001
**Hypertension, %**	42.4	78.3	<0.001
**Diabetes, %**	20.7	34.5	<0.001
**Smoking, %**	47.8	58.1	0.003
**Stroke, %**	5.7	7.5	0.323
**Ischemic heart disease, %**	10.5	12.5	0.331
**Medication**			
**ACEIs/ARBs usage, %**	8.6	14.6	0.005
**Statins usage, %**	6.8	5.7	0.586
**CCBs usage, %**	12.8	24.2	<0.001

* The cut-off refers to the corresponding cut-off point to each age group.

***BMI*** body mass index, ***SBP*** systolic blood pressure, ***DBP*** diastolic blood pressure, ***MAP*** Mean arterial pressure; ***BaPWV*** Brachial-ankle pulse wave velocity, ***CCBs*** Calcium channel blockers, ***ACEIs*** angiotensin converting enzyme inhibitors, ***ARBs*** angiotensin receptor blockers, ***HR*** heart rate, ***TC*** total cholesterol, ***LDL-C*** low density lipoprotein cholesterol, ***HDL-C*** high density lipoprotein cholesterol, ***TG*** triglyceride.

**Table 5:** Characteristics of female participants with vs. without high baPWV in the overall sample

**Variables**	Low baPWV baPWV < cut-off*	High baPWV baPWV > cut-off*	**p value**
**n**	1213	363	
**Age , years**	52.8 ±15.2	58.1 ± 13.2	<0.001
**baPWV, cm/s**	1441 ± 318	2038 ± 566	<0.001
**BMI, kg/m**^**2**^	23.9 ± 3.3	24.4 ± 3.7	0.026
**SBP, mmHg**	126.9 ± 19.5	144.2 ± 20.0	<0.001
**DBP, mmHg**	74.8 ± 10.5	82.5 ± 10.4	0.615
**HR, beats/min**	71 ± 9	75 ±11	<0.001
**TC, mmol/L**	4.99 ± 1.0	5.38 ±1.1	<0.001
**HDL-C, mmol/L**	1.41 ± 0.3	1.35 ±0.3	0.003
**LDL-C, mmol/L**	2.95 ± 0.9	3.19 ±0.9	<0.001
**TG, mmol/L**	1.51 ± 0.9	1.80 ± 1.1	<0.001
**Fasting glucose, mmol/L**	5.31 ± 1.5	5.89 ± 1.9	<0.001
**Hypertension, %**	40.1	73.3	<0.001
**Diabetes, %**	17.9	31.9	<0.001
**Smoking, %**	3.6	4.1	0.638
**Stroke, %**	3.6	6.3	0.058
**Ischemic heart disease, %**	6.8	7.9	0.485
**Medication**			
**ACEIs/ARBs usage, %**	7.0	15.2	<0.001
**Statins usage, %**	4.6	6.1	0.271
**CCBs usage, %**	11.2	25.3	<0.001

* The cut-off refers to the corresponding cut-off point to each age group.

***BMI*** body mass index, ***SBP*** systolic blood pressure, ***DBP*** diastolic blood pressure, ***MAP*** Mean arterial pressure; ***BaPWV*** Brachial-ankle pulse wave velocity, ***CCBs*** Calcium channel blockers, ***ACEIs*** angiotensin converting enzyme inhibitors, ***ARBs*** angiotensin receptor blockers, ***HR*** heart rate, ***TC*** total cholesterol, ***LDL-C*** low density lipoprotein cholesterol, ***HDL-C*** high density lipoprotein cholesterol, ***TG*** triglyceride.

Results of multiple backward stepwise logistic regression analyses in male and female population are shown in Table 6. Based on the entry criteria mentioned in the statistical analysis section, variables entering the multiple logistic regression analysis included age (per 1 year), SBP (per 1 mmHg), DBP (per 1 mmHg), HR (per 1 beat/min), fasting glucose (per 1 mmol/L), diabetes (yes/no), hypertension (yes/no), CCBs usage (yes/no), ACEIs/ARBs usage (yes/no), and smoking (yes/no) in men; while in women, the included variables were age (per 1 year), SBP (per 1 mmHg), BMI (per 1 kg/m^2^), HR (per 1 beats/min), TC (per 1 mmol/L), HDL-C (per 1 mmol/L), TG (per 1 mmol/L), fasting glucose (per 1 mmol/L), diabetes (yes/no), hypertension (yes/no), CCBs usage (yes/no), ACEIs/ARBs usage (yes/no). As shown in Table 6, variables positively associated with high baPWV were HR (OR = 1.029, [1.015, 1.044], p < 0.001), SBP (OR = 1.055, [1.045, 1.065], p < 0.001), fasting glucose level (OR = 1.023, [1.086 to 1.331], p < 0.001) and smoking (OR = 1.271, [0.944, 1.710], p = 0.032) in men; while in women, the variables were SBP (OR = 1.048, [1.040, 1.056], p < 0.001), diabetes (OR = 1.835, [1.299, 2.593], p = 0.001), TC levels (OR = 1.220, [1.076, 1.384], p = 0.002) and HR (OR = 1.044, [1.032, 1.056], p < 0.001).

**Table 6:** Associations between high baPWV* and potential risk factors based on the multiple logistic regression analyses†

**Variables‡**	**Male**		**Female**	
	**OR (95% CI)**	**p value**	**OR (95% CI)**	**p value**
**SBP, per 1 mmHg**	1.055 (1.045, 1.065)	< 0.001	1.048 (1.040, 1.056)	< 0.001
**HR, per 1 beats/min**	1.029 (1.015, 1.044)	< 0.001	1.044 (1.032, 1.056)	< 0.001
**TC, per 1 mmol/L**	─ ^a^	─	1.220 (1.076, 1.384)	0.002
**Fasting glucose, per 1 mmol/L**	1.203 (1.086, 1.331)	< 0.001	─	─
**Hypertension (yes/no)**	─	─	─	─
**Diabetes (yes/no)**	─	─	1.835 (1.299, 2.593)	0.001
**Smoking (yes/no)**	1.271 (0.944, 1.710)	0.032	─	─

* High baPWV was defined based on age-specific cut-off points.

† The backward stepwise method was used.

**‡** Only those that entered the final step of backward stepwise logistic regression were shown in the table. Originally, variables entering the multiple logistic regression model included age, SBP, DBP, HR, fasting glucose, diabetes, hypertension, CCBs usage, ACEIs/ARBs usage, and smoking in men; while in women, the included variables were age, SBP, BMI, HR, TC, HDL-C, TG, fasting glucose, diabetes, hypertension, CCBs usage, ACEIs/ARBs usage.

^a^ The symbol “─“ represents covariates that did not significantly contribute to the model or were not included.

***OR*** odds ratio, ***CI*** confidence interval, ***BMI*** body mass index, ***SBP*** systolic blood pressure, ***DBP*** diastolic blood pressure, ***CCBs*** Calcium channel blockers, ***ACEIs*** angiotensin converting enzyme inhibitors, ***ARBs*** angiotensin receptor blockers, ***HR*** heart rate, ***TC*** total cholesterol, ***HDL-C*** high density lipoprotein cholesterol, ***TG*** triglyceride.

## Discussion

Early detection of arterial stiffness is useful in primary and secondary prevention of a series of major CVD like coronary artery disease and hypertension. Several studies have shown that PWV was an independent predictor of future development of CVD [12,13]. The baPWV measurement is noninvasive and convenient, and has been used for cardiovascular risk stratification [14]. For the first time, the present study investigated the prevalence of arterial stiffness in North China on the basis of a 23-community-based study population, and explored the association between high baPWV and CVD risk factors based on general population other than highly characteristic cohorts. Also, the age-specific cut-off points of baPWV were determined and evaluated in terms of its associations with increased arterial stiffness defined by high baPWV in male and female northern Chinese population.

The present data indicated that males had a higher average and upper limit baPWV values than females. In the healthy reference aged 50–59 years, women had a higher age-specific cut-off value of arterial stiffness than men did. Previous researches have indicated that estrogen have beneficial effects on arterial stiffness. A Japanese study reported that the menopause augments the age-related increase in arterial stiffness during the early postmenopausal phase and that this augmentation is probably related, at least in part, to estrogen deficiency [15]. Also, Sztejnsznajd and et al. claimed that long-term administration of oral estrogens leads to an improvement in arterial distensibility in postmenopausal women [16]. The mean age of natural menopause in China was 48.7 years according to a recent study [17]. Therefore, we may infer that decrease of estrogen in menopause might take effect on the arterial stiffness.

The male participants in 50–59 years age group had the greatest prevalence of arterial stiffness, while it was the case in 60–69 years age group of female participants. Considering that arterial stiffness had been associated with the risk of CVD, the present result may suggest that the burden of CVD associated to arterial stiffness rise to the greatest extent in middle-age population. It should be pointed out that, were the cut-off points for high PWV defined irrespectively of age group, the prevalence of “high baPWV” would have gone up sharply with increasing age in both genders. However, results in this circumstance would be invalid due to the confounding effects of age. Two recent studies showed that higher baPWV was associated with an increased risk total mortality [18,19], and inferred that healthiest people with lower baPWV are more likely to survive past 60 years. This might contribute to a greater prevalence of high baPWV in middle-aged group. Follow-up of the present study is expected to further the interpretation.

In this study, the identified potential risk factors of arterial stiffness based on baPWV measurement were faster HR, higher SBP, higher fasting glucose level, and smoking in men, while were HR, SBP, TC level, and diabetes in women. Two previous studies observed the presence of increased arterial stiffness in patients with hypertension, dyslipidemia and/or diabetes [20,21]. The present data suggested that SBP should be an important clinical indicator of increased arterial stiffness both in men and in women.

Smoking is another major risk factor in the development and progression of CVD [22]. This study indicated that it may also be responsible for greater arterial stiffness, especially in men. Alterations in hemostatic factors and injury to the vascular endothelial function may play an important role in increasing arterial stiffness [23]. The reason why no significant association was found between smoking and baPWV in women is probably due to the fact that the small number of female smokers could not yield statistical significance.

The present data showed that the higher HR was significantly associated with the high baPWV in men and in women. HR changes can influence arterial distensibility [24]. An epidemiological study assessed the relationship between HR and PWV and demonstrated that there was a statistically significant positive link between the higher HR and the higher arterial stiffness [25]. It is worth further investigation that whether lowering HR therapy using β-blockers or other methods benefits patients with increased arterial stiffness or not. Obesity is also an established risk factor of CVD and arterial stiffness [26]. However, we found no significant relation between BMI and baPWV in multivariable models. This is partly due to the low prevalence of obesity in the population, which limits power to detect a relation.

The strength of this study is a multi-community-based general population with the large sample size, which makes it possible to stratify subjects by age group, and results generalizable to individuals in different ages. However, the present study has limitations. Firstly, response rate was 53.6%. A low response rate may give rise to sampling bias if the nonresponse is unequal among the participants regarding exposure and/or outcome. Though the most common reason was refusal of examinations, it was unclear that whether other reasons not to participate related to factors associated with arterial stiffness. Secondly, the present study did not examine markers of inflammation and renal function, and therefore, could not evaluate this particular relationship. Besides, the limitation of the cross-sectional design was also a fact in this study. The prognostic significance of the baPWV could not be evaluated. Finally, the population was from North China, and may not suitable applied for south areas of China and other countries. Longitudinal studies with a large sample size and a follow-up of the present study are expected to further explore these questions.

## Conclusions

The prevalence of arterial stiffness based on high baPWV was 22.30% in men and 26.40% in women respectively in the North China. On the basis of the age-specific cut-off points, the middle-age population had the highest prevalence of high baPWV. The factors associated with arterial stiffness were HR, SBP, fasting glucose level, and smoking in men, while TC, HR, SBP, and diabetes in women.

## Competing interests

The authors declare no conflict of interest.

## Authors’ contributions

The work presented here was carried out in collaboration between all authors. JW, ZZ and DH designed the research. JW and ZZ carried out the data collection, analyzed the data, and interpreted the results. JW and ZZ drafted the manuscript. All authors have contributed to, seen and approved the manuscript.

## Pre-publication history

The pre-publication history for this paper can be accessed here:

http://www.biomedcentral.com/1471-2261/12/119/prepub
